# Potential Biomarkers of Insulin Resistance and Atherosclerosis in Type 2 Diabetes Mellitus Patients with Coronary Artery Disease

**DOI:** 10.1155/2013/698567

**Published:** 2013-10-24

**Authors:** Sharifah Intan Qhadijah Syed Ikmal, Hasniza Zaman Huri, Shireene Ratna Vethakkan, Wan Azman Wan Ahmad

**Affiliations:** ^1^Department of Pharmacy, Faculty of Medicine, University of Malaya, 50603 Kuala Lumpur, Malaysia; ^2^Clinical Investigation Centre, 13th Floor Main Tower, University Malaya Medical Centre, 59100 Lembah Pantai, Kuala Lumpur, Malaysia; ^3^Endocrinology Unit, Department of Medicine, Faculty of Medicine, University of Malaya, 50603 Kuala Lumpur, Malaysia; ^4^Cardiology Unit, Department of Medicine, Faculty of Medicine, University of Malaya, 50603 Kuala Lumpur, Malaysia

## Abstract

Type 2 diabetes mellitus patients with coronary artery disease have become a major public health concern. The occurrence of insulin resistance accompanied with endothelial dysfunction worsens the state of atherosclerosis in type 2 diabetes mellitus patients. The combination of insulin resistance and endothelial dysfunction leads to coronary artery disease and ischemic heart disease complications. A recognized biological marker, high-sensitivity C-reactive protein, has been used widely to assess the progression of atherosclerosis and inflammation. Along with coronary arterial damage and inflammatory processes, high-sensitivity C-reactive protein is considered as an essential atherosclerosis marker in patients with cardiovascular disease, but not as an insulin resistance marker in type 2 diabetes mellitus patients. A new biological marker that can act as a reliable indicator of both the exact state of insulin resistance and atherosclerosis is required to facilitate optimal health management of diabetic patients. Malfunctioning of insulin mechanism and endothelial dysfunction leads to innate immune activation and released several biological markers into circulation. This review examines potential biological markers, YKL-40, alpha-hydroxybutyrate, soluble CD36, leptin, resistin, interleukin-18, retinol binding protein-4, and chemerin, as they may play significant roles in insulin resistance and atherosclerosis in type 2 diabetes mellitus patients with coronary artery disease.

## 1. Review

In 2010, it was estimated that 285 million people had been diagnosed with diabetes mellitus worldwide, a prevalence of 6.4%. This is predicted to increase to 439 million, a prevalence of 7.7%, by 2030 [1]. The number of deaths indirectly linked to diabetes mellitus is estimated to be 3.96 million per year for all age groups, a prevalence of 6.8% [2]. Type 2 diabetes mellitus (T2DM) (previously known as non insulin-dependent diabetes mellitus) accounts for about 90% of diabetic patients worldwide [1]. T2DM is described as a “silent disease” and is characterized by the combination of inadequate insulin secretion due to islet *β*-cell deterioration and insulin resistance [3]. T2DM is an independent risk factor for the development and mortality of various complications, implicate microvascular problems including retinopathy, neuropathy, and nephropathy; and macrovascular problems including coronary artery disease (CAD) [4, 5].

CAD is one of the most common causes of death among diabetic patients, with a 2- to 3-fold higher prevalence compared with nondiabetic people [2]. CAD is defined conservatively as past myocardial infarction (MI), coronary artery bypass graft (CABG), or percutaneous transluminal coronary angioplasty through confirmation by review of medical records or a major Q wave on electrocardiogram examination (ECG) in Insulin Resistance Atherosclerosis Study (IRAS) [6]. Concomitant risk factors, including persistent hyperglycemia, dyslipidemia, hypertension, smoking, a family history of the disease, and the presence of micro- and macroalbuminuria, directly increase the mortality risk of CAD among T2DM patients by approximately 2- to 4-fold compared with nondiabetic people [4, 5]. CAD is defined as an accumulation of cholesterol substance build-up in coronary arteries. Anatomically, coronary arteries are blood vessels supplying oxygen to cardiac muscle. Coronary arteries branch off from the aorta into four major arteries. These are the right coronary artery, the left main coronary artery, the left anterior descending artery, and the left circumflex artery. Blockage to any of these arteries due to plaque instability, known as atherosclerosis, leads to angina and ischemic conditions, resulting in CAD and also increasing the potential development of ischemic heart disease and other major cardiovascular diseases (CVDs) [7].

Owing to recent advances in understanding circulating molecular actions between endothelial function and the immune system, several potential biomarkers have been identified that appear to be linked to T2DM and CAD in terms of insulin resistance and atherosclerosis. This review examines these potential biomarkers as a new alternative to determining the status of insulin resistance in T2DM patients with CAD.

## 2. Mechanism of Insulin Resistance

According to the American Diabetes Association 2013 guidelines, insulin resistance and persistent hyperinsulinemia are found in a variety of medical conditions, including dyslipidemia and hypertension [4, 5]. Insulin resistance has been established as a precursor and acts as a strong factor linking T2DM with CVD. Studies have reported an increased potential risk of diabetic patients acquiring CAD within the past two decades [2, 6]. This is mainly because of insulin resistance development through genetics and environmental factors [8]. Alterations of *β*-cell function and insulin properties underlay the metabolic syndrome that includes dyslipidemia, hyperglycemia, hypertension, impaired fibrinolysis, and atherosclerosis, thus contributing to lower insulin sensitivity [6].

**Figure 1:**
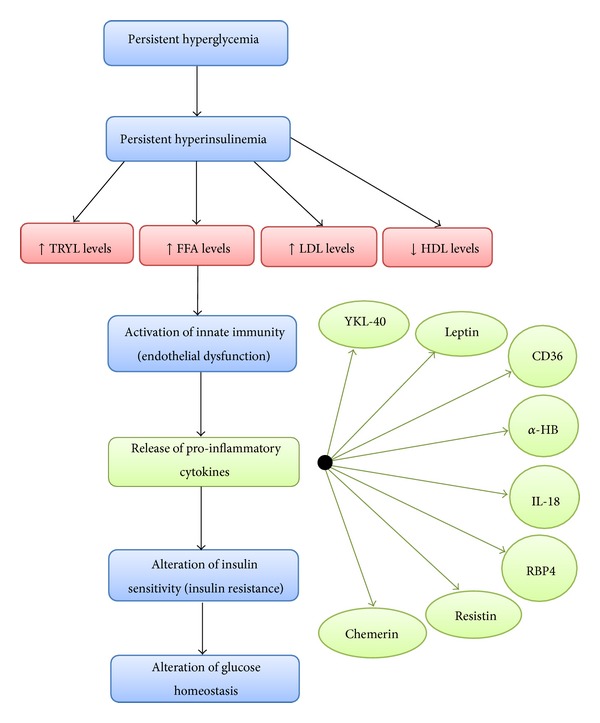
Mechanism of insulin resistance, endothelial dysfunction and proinflammatory secretion.

At a basic level, insulin resistance occurs through the activation of various types of macrophages. Malfunctioning adipocytes and adipose tissue release greater amounts of various proinflammatory cytokines. Subclinical inflammation due to insulin resistance might also correlate with the pathogenesis of all phases of atherosclerosis. This involves low-grade elevation of acute phase reactants, proinflammatory cytokine secretion, and cell adhesion molecules and leads to myocardial infarction, stroke, and other major peripheral vascular diseases. These factors, therefore, increase cardiovascular mortality. Activation of the innate immune system greatly contributes to the occurrence of T2DM and CAD (Figure 1) [9].

Endothelial dysfunction triggered by persistent inflammation due to increased levels of triglycerides (TRYL), free fatty acids (FFA) and low-density lipoprotein (LDL), and decreased levels of high-density lipoprotein (HDL) that eventually leads to alteration of insulin signaling and glucose uptake in muscles and adipocytes. 

Inefficient glucose uptake by muscle and adipose tissue leads to insulin resistance or a medical condition known as compensatory hyperinsulinemia. *β* cells of the pancreatic islets release excessive amounts of insulin in an attempt to compensate for high plasma glucose levels in the blood [9, 10]. Persistent hyperinsulinemia increases serum levels of triglycerides, free fatty acids (FFA), and low-density lipoprotein and decreases serum levels of high-density lipoprotein. Increased levels of circulating FFA in the blood activate the innate immune system to release pr-inflammatory cytokines, including tumor necrosis factor-*α*, interleukin (IL)-6, and IL-1*β* [11]. The presence of these cytokines leads to an alteration in insulin sensitivity and disruption of glucose homeostasis [11]. The process involves cytokines first mediating insulin signaling mechanisms in adipocytes, muscles, and the liver to increase the occurrence of insulin resistance [11], before disabling liver X receptors (LXRs) causing an increase in the accumulation of cholesterol, thus stimulating hepatic production and secretion of inflammatory markers, including C-reactive protein, plasminogen inhibitor-1, serum amyloid-A, *α*1-acid glycoprotein, and haptoglobin [12]. Cytokines then stimulate fibrinogen, which acts as an atherosclerotic risk factor and leads to CAD [11]. Finally, cytokines increase production of very low-density lipoprotein and FFA, which leads to the characteristics of diabetic dyslipidemia and the subsequent increase in plaque accumulation [9, 11, 12].

## 3. Correlation between Insulin Resistance, Endothelial Dysfunction, and Atherosclerosis

The endothelium is located at the interior surface of blood and lymphatic vessels. The endothelium consists of a thin layer of cells defined as being either vascular endothelial cells (cells in direct contact with blood) or lymphatic endothelial cells (cells in direct contact with lymph). The function of the endothelium is to sense mechanical stimuli, such as high pressure and stretching, and hormonal stimuli, such as vasoactive substances. The endothelium plays a role in the regulation of vasomotor functions, stimulates inflammatory processes, and influences hemostasis [13].

Studies suggest that persistent hyperinsulinemia might trigger endothelial dysfunction. In diabetes, the occurrence of insulin resistance is due to an alteration in insulin signaling. Once this alteration happens, phosphorylation of major pathways (e.g., phosphatidylinositol 3-kinase, phosphoinositide-dependent kinase-1, and AKT/protein kinase B pathways), which activate endothelial nitric-oxide synthase (eNOS), is downregulated drastically. Due to this downregulation, the role of eNOS changes from an antiatherogenic effect to a proatherogenic effect, which further contributes to the development of atherosclerosis. One of the insulin receptor pathways, mitogen-activated protein kinase, which stimulates mitogenic effects and growth, remains unaffected [13, 14].

**Figure 2:**
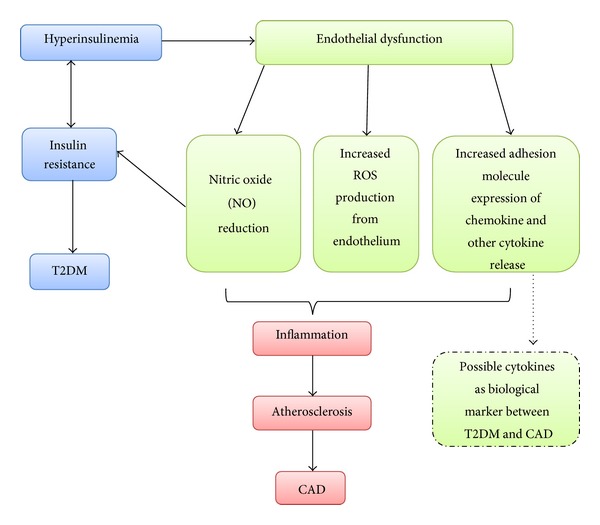
Insulin resistance in T2DM and endothelial dysfunction in CAD development.

A common mechanism of endothelium dysfunction is the depletion of eNOS. According to Willa and Manuel 2003, prolonged decreases of eNOS lead to decreased bioavailability of nitric oxide (NO), which acts as vascular protection by inhibiting inflammation, oxidation, vascular smooth muscle cell proliferation, and migration [14]. Decreased bioavailability of NO, along with low levels of high-density lipoprotein, high levels of small, dense, low-density lipoprotein, high secretion of angiotensin II sensitivity, and high releases of FFA in the blood, worsen is the status of endothelial dysfunction and promotes further atherogenic processes [14]. In addition, the effects of inflammation and reactive oxygen species contribute to decreasing NO bioavailability and stimulate secretion of proinflammatory cytokines, which are termed as possible biomarkers. Identification of these biomarkers might serve as tools for predicting insulin resistance and endothelial dysfunction in T2DM patients with CAD (Figure 2) [14].

The persistent events of hyperinsulinemia lead to insulin resistance and results to T2DM. Frequent hyperinsulinemia due to increased level of triglycerides, FFAs and LDL and decreased HDL, contributes to endothelial dysfunction and interrupts nitric oxide (NO) secretion, increases reactive oxygen species (ROS) and free radicals formation, and interruptions of adhesion molecule expression of chemokine and cytokine release. All the mechanisms contribute to inflammation, atherosclerosis, and CAD. Several cytokines had been discovered and used as biomarkers, strongly supporting the idea that the occurrence of hyperinsulinemia correlates with endothelial dysfunction leading to major diseases, T2DM, and CAD.

## 4. Potential Biomarkers for Insulin Resistance in T2DM Patients with Coronary Artery Disease

### 4.1. YKL-40

YKL-40 or alternatively termed as BPR-39 or human cartilage glycoprotein-39, produced by the gene Chitinase 3-like 1 (CH3L1) [15], is a heparin- and chitin-binding lectin without chitinase activity and a member of the mammalian chitinase-like protein cluster [16]. YKL-40 belongs to the glycosyl hydrolase family 18 which consists of enzymes and proteins, includes hydrolytic enzymes named as chitinases from various species including mammalian, bacteria, fungi, nematodes, insects and plants [16]. YKL-40 based on its three NH_2_-terminal amino acids, tyrosine (Y), lysine (K), and leucine (L) and its molecular weight of 40 kDa [16], is located at chromosome 1q31-q32 [15], consists of 10 exons and spans about 8 kb of genomic DNA [16], and has a crystal structure [17]. YKL-40 is produced at the site of inflammation [18], secreted by activated macrophages, including activated neutrophils, arthritic chondrocytes, fibroblast-like synovial cells, osteoblasts, and differentiated vascular smooth muscle cells [15].

Even though minor research has been conducted on the exact functions of YKL-40, several studies have reported that YKL-40 is an essential factor in extracellular tissue remodeling involving type 1 collagen fibril formation, a growth factor for fibroblasts and chondrocytes, and also controls mitogenesis by modulating MAP kinase and PI-3 K signaling cascades in fibroblasts [16, 19]. YKL-40′s association with migration, reorganization, and adhesion of vascular endothelial cells and vascular smooth muscle cells suggests that it may also play a role in angiogenesis [16, 19].

YKL-40 serum levels increase in patients with acute infections [18] and chronic inflammation [15]. Recent studies have reported that elevated levels of plasma YKL-40 are proportional with the HOMA-IR in T2DM subjects. This indicates that YKL-40 shows some correlation with insulin resistance and dyslipidemia. High levels of YKL-40 also appeared in adult subjects who did not report a medical history of T2DM and CVD comorbidities [20, 21], even at the childhood stage [22]. Other studies have reported elevated levels of plasma YKL-40 and albuminuria detected in both type 1 and type 2 diabetes mellitus patients [21]. The results of these studies suggest that YKL-40 might act as a potential biomarker for endothelial dysfunction, atherosclerosis, insulin resistance, and T2DM [20].

### 4.2. Alpha-Hydroxybutyrate

Alpha-hydroxybutyrate (*α*-HB) has been found to be the most significant biomarker associated with insulin sensitivity, diabetes mellitus, and CVD. According to Walter et al. 2010, the underlying biochemical mechanisms of alpha-hydroxybutyrate involve lipid oxidation and oxidative stress [23]. Alpha-hydroxybutyrate acts as an earlier marker of dysglycemia when compared to other biomarkers in the same research, such as alpha-ketobutyrate (*α*-KB), creatine, acylcarnitines, and lysoglycerophospholipids [23]. The study showed that the expression of alpha-ketobutyrate served as early indicator in insulin resistance by differentiating the group of normal glucose tolerance-insulin sensitivity (NGT-IS) from normal glucose tolerance-insulin resistance (NGT-IR) among nondiabetic population [23].

Alpha-hydroxybutyrate is an organic acid that is formed as a by-product during production of alpha-ketobutyrate through a reaction catalyzed by lactate dehydrogenase (LDH) or by an LDH isoform in the heart known as alpha-hydroxybutyrate dehydrogenase (*α*-HBDH) [23, 24]. Elevated levels of alpha-hydroxybutyrate occur due to an increased rate of alpha-ketobutyrate catabolism or inhibition of the products of dehydrogenase that catalyze the conversion of alpha-ketobutyrate to propionyl-CoA [25].

Walter et al. 2010 also reported that elevated levels of alpha-hydroxybutyrate might be associated with insulin resistance by two possible mechanisms [23]. First, the increment of hepatic glutathione stress causes increased production of glutathione, which contributes to the supply of more alpha-ketobutyrate substrate and subsequently results in increased formation of alpha-hydroxybutyrate. Second, increased levels of lipid oxidation lead to increased levels of nicotinamide adenine dinucleotide (NADH or NAD+), are parallel to the concentration of insulin-inhibited free fatty acid (FFA) [25]. Previous study showed positive correlation between steady states of FFA and plasma alpha-hydroxybutyrate in the diabetic cohorts. This supports the idea that increasing amounts of NADH or NAD+ correlate with the reduction of alpha-ketobutyrate to alpha-hydroxybutyrate [23].

### 4.3. Soluble CD36

CD36 also known as Fatty Acid Translocase (FAT) is a complex multifunctional protein that present as mononuclear phagocytes, serves as a scavenger receptor for oxidized low-density lipoprotein (LDL), cellular transporter of long chain fatty acids in muscles and adipocytes, and apoptotic cells on macrophages [26, 27]. CD36 has been also showed to be involved in several processes, including long-chain fatty acids, advanced glycosylation products, oxidized phosphocholines, collagen, growth-hormone releasing hormone (GNRH), peptides hexarelin and thrombospondin-1 (TSP-1) [28].

In 2008, Handberg et al. studies' has identified the existence and availability of soluble CD36 in cell-free plasma for further research discovery [29]. CD36 has been proposed as a biomarker of macrophage activation and inflammation [30] and atherosclerosis [31]. Several studies report that the expression of this 88 kDa transmembrane glycoprotein CD36 is strongly associated with atherosclerosis, angiogenesis, inflammation, lipid metabolism, platelet activation [31–33], hyperglycemia, and insulin resistance [34]. Oxidized LDL stimulates membrane CD36 expression on the surface of monocytes and macrophages, resulting in an increased atherosclerotic effect, and might be the underlying mechanism causing lipid accumulation in the subendothelial space [27, 30–32]. 

Previous study discovered that CD36 has the ability to bind and modify LDL that is trapped in arterial wall, contributing to the formation of lipid-engorged macrophage foam cells and initiate atherosclerotic lesions [26, 27, 31]. CD36 interaction with oxidized LDL stimulates a signaling response that act as proinflammatory and proatherogenic where they differentiate into macrophages [26]. The signaling pathway involves activation of Src-family kinases and MAP kinases and Vav family guanine nucleotide exchange factors, thereby contributing to ligand internalization, foam cell formation, and inhibition of migration [26]. 

Activation mechanism of CD36 pathway started when LDL particles cross the endothelium and become trapped in the intima connective tissue [26]. Under the influence of proinflammatory cytokines, the macrophage produces reactive oxygen and nitrogen species which oxidized the unsaturated phospholipids present in LDL. One oxidized, the LDL particles lose their infinity to its specific LDL receptor but gain affinity for scavenger receptors, including CD36, and internalized by intima macrophages [26]. During internalization, specific oxidized lipids present in oxidized LDL serve as ligands or precursors of ligands for the nuclear hormone receptor peroxisome proliferator-activated receptor *γ*-dependent (PPAR-*γ*). This receptor, once engaged and activated, acts as a transcription factor that drives disorderly expression of many metabolic genes, including CD36 [26].

Furthermore, elevated FFA in monocytes and macrophages also stimulate CD36 expression through PPAR-*γ*-dependent mechanism [26, 31, 33]. Fat accumulation in the human liver results in elevated levels of FFA and lipolysis eventually leads to insulin resistance and diabetic dyslipidemia. Upregulation of CD36 expression in insulin-resistant subjects, which involves an impaired insulin signaling cascade, is another pathological mechanism [30].

Increased levels of CD36 in monocytes among diabetic patients are highly correlated with insulin resistance. Several studies had showed that high levels of CD36 present in pre-diabetes, overt diabetes, polycystic ovary syndrome (PCOS), and impaired glucose tolerance strongly suggest that CD36 is involved in diabetes [30] and atherosclerosis pathogenesis and acts as inflammation biomarker [31, 32].

### 4.4. Leptin

Plasma leptin has a strong correlation with obesity, T2DM, CVD, insulin resistance, metabolic syndromes, and inflammatory markers [35]. Leptin, a 16 kDa hormone component of adipokine, is stimulated and secreted specifically by white adipose cells [36] and has been proposed as a biomarker for atherosclerotic CVD [37]. The expression of leptin receptors, mainly in atherosclerotic lesions [38], is involved in a variety of actions, including endothelial activation [39, 40], smooth muscle cell proliferation and calcification [41], and activation of monocytes and adaptive immune responses [42]. Studies have reported that leptin levels are linked to inflammatory and fibrinolysis markers, including C-reactive protein and plasminogen activator inhibitor-1, and are associated with CVD [35, 43].

Leptin is an adipocyte-specific *ob* gene product that has been found to be associated with insulin resistance and diabetes in obesity patients through insulin sensitivity and insulin secretion alterations [44]. Low insulin sensitivity has a pathophysiological effect on metabolic syndromes, including central obesity, dyslipidemia, hyperglycemia, hypertension, impaired fibrinolysis, and atherosclerosis [43]. Leptin secretion by adipocytes might be stimulated by insulin, which directly influences islet *β*-cell action on insulin levels [45]. Recent research reported a significant association between leptin in coronary heart disease (CHD) and insulin resistance [46].

### 4.5. Resistin

Resistin and resistin-like molecule protein originates from a family of cysteine-rich secretory proteins produced during adipocyte catabolism in the presence of the thiazolidinediones, a cluster of insulin-sensitizing drugs [36]. Resistin plays a role in the regulation of energy, glucose, and lipid homeostasis [47] and the maintenance of fasting blood glucose levels [48] by modulating hepatic insulin action [37, 49]. Resistin is a macrophage-derived signaling polypeptide hormone with a molecular weight of 12.5 kDa and is 108 amino acids long. It has low circulating levels [50], but in some studies, it has been reported to be upregulated in insulin resistance, T2DM, and CVD [51].

The expression of resistin primarily by monocytes and macrophages is much greater compared with adipocyte catabolism [47]. Resistin may increase the susceptibility of metabolic syndrome (MS) by regulating adiponectin secretion from adipocytes and enhancing hepatic gluconeogenesis by inhibiting the enzymes involved in gluconeogenesis through AMP-activated protein kinase activation [52]. A recent study reported that subjects with premature atherosclerosis have higher levels of plasma resistin compared with subjects with established atherosclerosis [53]. Correlations between insulin sensitivity, adiposity, and T2DM [54] still remain to be fully revealed, even though resistin has been proposed as a potential link between obesity, insulin resistance, and T2DM with CVD [51, 55].

### 4.6. Interleukin-18

The proinflammatory cytokine, IL-18, is located on chromosome 11q22.2-22.3 [56] and is a member of the IL-1 cluster. Originally, IL-18 was described as an interferon-*γ*-inducing factor because of its strong ability to stimulate interferon-*γ* release with the presence of co-stimuli, such as IL-12 or lipopolysaccharide [57]. Recently, studies have suggested that IL-18 is involved in apoptosis and tissue destruction [58], as part of the host defense against infections and neoplasms [57], abnormally expressed in adipose tissue through a mechanism called lipodystrophy [59], and is a predictor of cardiovascular mortality among coronary atherosclerosis subjects [60].

Inflammatory activity by macrophages, monocytes, dendritic cells, osteoblastic stroma cells, and cells of the central nervous system (CNS) stimulates activity of the precursor of IL-18, called pro-IL-18. Pro-IL-18 is cleaved by either caspase-1-dependent conversion [61] or through the FAS ligand, in a caspade-1-independent processing of IL-18 manner [62], to release the active peptide [63]. Once secreted, the active peptide of IL-18 can bind to either the IL-18 receptor or IL-18 binding protein or might be bind to the both. The IL-18 receptor consists of an *α*-chain and a *β*-chain. The *α*-chain is responsible for extracellular binding of IL-18 and the *β*-chain is responsible for intracellular signal transduction [64]. As for controlling proinflammatory activity, excess amounts of IL-18 secretion will bind to IL-18 binding protein, which result in a free fraction of IL-18 in a negative feedback mechanism. This free fraction of IL-18 is able to activate the *β*-chain while combination of free fraction of IL-18 and protein-bound IL-18 is able to activate the *α*-chain [65]. 

The general mechanisms of IL-18 in the context of insulin resistance involve lineal effects of IL-18 on insulin signaling with or without tumor necrosis factor-*α* (TNF-*α*) stimulation in tissues and a secondary response of IL-18 to insulin resistance. Previous research has reported a slight correlation between IL-18 and fasting plasma glucose in T2DM [66] and between IL-18 and fasting plasma insulin in obese women [67]. Persistent circulatory levels of IL-18 have been reported in T2DM subjects in parallel with elevated fasting glucose levels and hyperglycemia [67]. However, IL-18 appears to act as an indicator for insulin resistance but not for *β*-cell malfunction. It has therefore been suggested that there is a plausible correlation between the functions of IL-18 and type 1 diabetes mellitus (T1DM) [56], T2DM [68], obesity [66], and CVD [60]. Recent investigation showed a strong and clear correlation between IL-18 and insulin resistance in T2DM subjects and also in non-T2DM subjects [68]. 

### 4.7. Retinol Binding Protein-4

Retinol binding protein-4 (RBP4) had been identified as the only specific transport protein for retinol (vitamin A) that delivers retinol to tissues from the blood [69]. It is highly expressed in adipose tissue compared with the liver and has a strong association with endothelial function. Research has reported that the region near the RBP4 locus on human chromosome 10q has been linked to an increased risk of T2DM [69]. Fischer et al. reported that decreasing secretion of RBP4 serum levels improved insulin action and showed strong associations between high RBP4 serum levels and insulin resistance [69]. Previously, RBP4 serum levels were found to have an association with insulin sensitivity and to increase in lean and obese, nondiabetic [70], and T2DM subjects [71]. 

Lack research had been done to discover the exact role of RBP4 in human metabolism as most research has been conducted using glucose transporter-4 knockout mice in an attempt to discover the mechanisms of RBP4 in adipose tissue. However, a recent study reported a strong correlation between RBP4 and insulin resistance in nondiabetic subjects without a medical or family history of diabetes [71].

Investigation showed that high secretion of RBP4 by adipocytes decreased the expression of glucose transporter-4 (GLUT-4) in adipose tissue, which is commonly found in T2DM [71]. A study reported that high circulating levels of serum RBP4 increased the potential for insulin resistance by blocking insulin signaling in muscle, thus increasing hepatic glucose output. However, correlations between RBP4 and vascular endothelium, oxidative stress, low-grade inflammation related to insulin resistance, and diabetic complications are still unclear [71].

### 4.8. Chemerin

Chemerin (also known as retinoic acid receptor responder 2 and tazarotene-induced gene 2) discovered as 18 kDa adipokine is secreted in the liver, acts as chemotactic agents and is highly stimulated by the innate immune system such as plasmacytoid dendritic cells and macrophages [72]. Chemerin had been discovered as a natural ligand of the chemerin receptor termed as ChemR23, also known as chemokine-like receptor 1 (CMKLR1). Chemerin also involves in intracellular calcium release and phosphorylation of extracellular signal-regulated kinase-1 and -2 (ERK 1/2) [72]. 

Chemerin is released as an inactive precursor. Through proteolytic cleavage, chemerin is activated by serine proteases of the coagulation, fibrinolytic, and inflammatory cascades. Chemerin is produced as a preproprotein, preprochemerin, which requires N-terminal cleavage of a secretion signal peptide before it is secreted as an inactive precursor protein, prochemerin. This proprotein has low biological activity and requires further extracellular C-terminal processing by plasmin, carboxypeptidases or serine proteases of the coagulation, fibrinolytic and inflammatory cascades [73]. 

These findings showed that chemerin has multiple cleavage sites in the C-terminal domain. In order to reach its maximal anti-inflammatory effects, bioactivity of chemerin is dependently regulated by proteolytic cleavage in the C-terminal region [74]. The presence of chemerin isoforms in hemofiltrate, serum, or ascites has potent chemotactic activity, indicating a proteolytic activation mechanism of chemerin bioactivity. Through mass spectrometry analysis, the isoforms of chemerin have been identified as chem21–154 and chem21–157, respectively; however, the proteases required for the isoforms activation remain unknown [74]. 

Increased activity of the coagulation cascade and decreased activity of the fibrinolytic cascade have been reported in obesity [75] and T2DM [76, 77], thus indicating the role of chemerin in immune responses [73]. The greater the activity of the serine proteases involved in coagulation, the higher the levels of activated chemerin [78].

A previous study has reported that chemerin is secreted equally in normal and T2DM subjects [77]. In another study, chemerin levels were reported as an independent biomarker of metabolic syndrome [79]. A recent study by Johanna et al. in 2010 reported that chemerin levels correlated with body mass index and waist-to-hip ratio but not with high-density lipoprotein cholesterol, which is highly secreted in obese and T2DM subjects. In terms of a biomarker, elevated levels of chemerin positively correlated with elevated levels of C-reactive protein in overweight and T2DM subjects [78].

## 5. Summary

**Figure 3:**
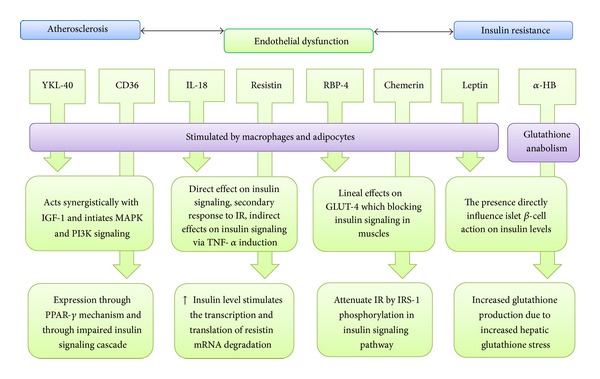
Summary of potential biomarkers mechanism.

The association between the pathophysiology of T2DM and CAD and the presence of biomarkers was summarised in Figure 3. Through endothelial dysfunction along with insulin resistance mechanism, different bioreceptors released biomarkers into the blood circulation to give a signal on the occurrence of inflammation. The presence of potential biomarkers might reflect an underlying disease pathophysiology which would be essential to predict future events and treatment response indication.

## 6. Conclusion

Studies have reported strong evidence that suggests YKL-40, *α*-HB, soluble CD36, leptin, resistin, IL-18, RBP4, and chemerin could be new biomarkers for the pathogenesis of insulin resistance and endothelial dysfunction in T2DM patients. Components of these biological markers have been proposed to act as predictors of cardiovascular events in diabetic patients. However, the exact role of these biomarkers in insulin resistance and associations between biomarkers and disease need to be further elucidated. It is important to have a detailed understanding of the involvement of these biomarkers to clarify the biological action of cytokines and endothelial dysfunction and the occurrence of insulin resistance. In conclusion, these potential biomarkers might provide an alternative diagnostic tool for ensuring optimal management of T2DM patients with CAD.

## Abbreviations

CAD: Coronary artery disease
CMKLR1: Chemokine-like receptor 1
CVD: Cardiovascular disease
FAT: Fatty Acid Translocase
eNOS: Endothelial nitric-oxide synthase
FFA: Free fatty acid
GLUT-4: Glucose Transporter 4
GNRH: Growth-hormone releasing hormone
HOMA-IR: Homeostasis model of assessment-insulin resistance
IGF-1: Insulin growth factor-1
IL: Interleukin
IR: Insulin resistance
IRIS: Insulin Resistance Atherosclerosis Study
IRS-1: Insulin receptor substrate-1
LDL: Low-density lipoprotein
MAPK: Mitogen-activated protein kinase
MS: Metabolic syndrome
NADH: Nicotinamide adenine dinucleotide
PI-3K: Phosphatidylinositol 3-kinase
PPAR-*γ*: Peroxisome proliferator-activated receptor *γ*-dependent
RBP4: Retinal binding protein-4
ROS: Reactive oxygen species
TNF-*α*: Tumor necrosis factor-*α*
T1DM: Type 1 diabetes mellitus
T2DM: Type 2 diabetes mellitus
TSP-1: Thrombospondin-1.
